# Horner syndrome following endoscopic thyroid surgery (ETS): a report of two cases and literature review

**DOI:** 10.3389/fmed.2025.1614914

**Published:** 2025-08-18

**Authors:** Tian-Hao Xie, Yan Fu, Xiao-Shi Jin, Si-Ning Ha, Xiang-Xiang Ren, Xin-Li Sun, Zheng Niu

**Affiliations:** ^1^Department of General Surgery, Affiliated Hospital of Hebei University, Baoding, Hebei, China; ^2^Basic Research Key Laboratory of General Surgery for Digital Medicine, Affiliated Hospital of Hebei University, Baoding, Hebei, China; ^3^Department of Ophthalmology, Baoding No.1 Central Hospital, Baoding, Hebei, China

**Keywords:** thyroid, horner syndrome, endoscopic thyroid surgery, complication, case report

## Abstract

Horner syndrome (HS), a rare complication of endoscopic thyroid surgery (ETS), manifests as ptosis, miosis, and anhidrosis resulting from oculosympathetic pathway disruption. This study explores HS etiology through two case reports and literature analysis. Case 1 involved a 43-year-old female who underwent unilateral thyroidectomy via a bilateral areolar approach for a thyroid oncocytic adenoma. On postoperative day 1, ptosis and miosis were observed, and the patient was diagnosed with HS. Despite initial glucocorticoid and neurotrophic therapy, symptoms resolved spontaneously by 6 months. Case 2 involved a 36-year-old female with papillary thyroid carcinoma treated via ETS with central lymph node dissection. Transient ptosis and miosis occurred postoperatively and resolved completely after a 6-day course of steroid treatment. Both cases highlighted HS as a complication linked to intraoperative cervical sympathetic chain (CSC) injury, likely due to retractor-induced compression, thermal damage from energy devices, or anatomical variations. A literature review identified only nine prior ETS-related HS cases, emphasizing its rarity (incidence: 0.03%–0.48%). Mechanisms include CSC compression caused by hematoma, edema, or inflammation in confined surgical spaces, with most symptoms resolving as these subside. Differential diagnosis requires excluding intracranial, spinal, or vascular pathologies. Pharmacologic tests utilizing drugs such as Apraclonidine, Cocaine, and Hydroxyamphetamine aid in the diagnosis of HS, while short-term use of steroids and neurotrophins may expedite recovery. Persistent HS beyond 1 year diminishes the likelihood of recovery, necessitating surgical correction for ptosis. ETS, favored for cosmetic outcomes, demands meticulous CSC preservation during dissection, particularly near the superior cervical ganglion. Preoperative patient counseling about HS risk is crucial. This study underscores HS as non-life-threatening yet distressing complication, advocating for refined surgical techniques and heightened anatomical awareness to avoid CSC injury during ETS.

## Introduction

Horner syndrome (HS), initially documented in 1869 by Swiss ophthalmologist Johann Friedrich Horner (1), represents a clinical condition stemming from the paralysis of the oculosympathetic pathway (OSP). Its characteristic “triad” of symptoms comprises ptosis, miosis, and anhidrosis. The primary etiologies of HS encompass tumors in the head and neck area, trauma, brainstem hemorrhage, brainstem infarction, myelitis, carotid artery dissection, infections, surgical procedures, and a multitude of other factors (2).

Endoscopic techniques have been applied in thyroid surgery for approximately three decades. In Gagner (3) introduced the pioneering technique of endoscopic subtotal parathyroidectomy. Subsequently, Hüscher et al. (4) were the first to successfully perform an endoscopic thyroid surgery (ETS) for right lobe adenoma. Since the first case of minimally invasive video-assisted thyroidectomy (MIVAT) for papillary thyroid microcarcinoma was reported by Micooli et al. (5), the indications for ETS have been further expanded, and ETS has gradually gained popularity in clinical practice. Additionally, various approaches of ETS have been subsequently reported. In Kang et al. (6) reported 200 cases of robot-assisted endoscopic thyroidectomy (RAET) for thyroid malignancies via a gasless transaxillary approach, offering another practical treatment option for patients with thyroid neoplasms.

ETS demonstrates better surgical outcomes in terms of aesthetic results compared to traditional open surgery, making it a preferred choice for young patients with thyroid neoplasms (7). However, HS, as a rare complication of ETS, causes significant cosmetic and psychological distress to patients. HS has been reported not only in ETS but also, rarely, in open thyroidectomy, especially during central lymph node dissection (8). Recognizing HS as a potential postoperative issue is crucial in clinical practice to ensure timely diagnosis and management. HS as a complication of ETS is extremely rare, with only 9 cases, which was accompanied by detailed clinical information, reported in previous literature (9–15), including 3 cases of MIVAT, 5 cases of ETS, and 1 case of RAET (Table 1). Herein, we report two cases of HS as a complication after ETS via the bilateral areolar approach, and conducted an analyze this rare complication in combination with previous literature.

**TABLE 1:** Reported cases of horner syndrome related to endoscopic thyroid surgery.

References	Histopathology	Approach	Extent of operation	Time of occurrence	Symptoms	Other complications	Special treatment	Follow-up time	Recovery outcome
Ying et al. (9)	PTC	MIVAT	UT + CLND	POD 2	Ptosis, miosis	None	Steroid	4 months	Incomplete resolved
	PTC	MIVAT	UT + CLND	POD 3	Ptosis, miosis	None	None	5 days	Resolved
Meng et al. (10)	PTC	ETS	UT + CLND	POD 1	Ptosis, miosis	None	Mecobalamin	11 months	Resolved
	PTC	ETS	UT + CLND	POD 3	Ptosis, miosis	None	None	1 months	Resolved
Hu et al. (11)	PTC	MIVAT	UT + CLND + LLND	POD 2	Ptosis, miosis, enophthalmos	None	Dexamethasone, mecobalamin	1 year	Incomplete resolved
Min et al. (12)	PTC	ETS	TT + CLND	POD 3	Ptosis, miosis, anhidrosis	None	Mecobalamin, vitamin B1	3 months	Resolved
Lee et al. (13)	PTC	RAET	TT	SD	Ptosis, miosis	None	None	12 months	No improvement
Xie et al. (14)	PTC	ETS	UT + CLND	POD 1	Ptosis, miosis, anhidrosis,	None	Dexamethasone, mecobalamin	6 months	Resolved
Chen et al. (15)	PTC	ETS	UT + CLND	SD	Ptosis, anhidrosis	None	None	6 weeks	Resolved
Personal cases	Oncocytic adenoma	ETS	UT	POD 1	Ptosis, miosis, anhidrosis	None	Dexamethasone, mecobalamin	6 months	Resolved
	PTC	ETS	UT + CLND	POD 1	Ptosis, miosis, anhidrosis	None	Dexamethasone, mecobalamin	6 days	Resolved

PTC, papillary thyroid carcinoma; MIVAT, minimally invasive video-assisted thyroidectomy; ETS, endoscopic thyroid surgery; RAET, robotic-assisted endoscopic thyroidectomy; UT, unilateral thyroidectomy; TT, total thyroidectomy; CLND, central lymph node dissection; SD, surgery day; POD, postoperative day.

### Case 1

**FIGURE 1:**
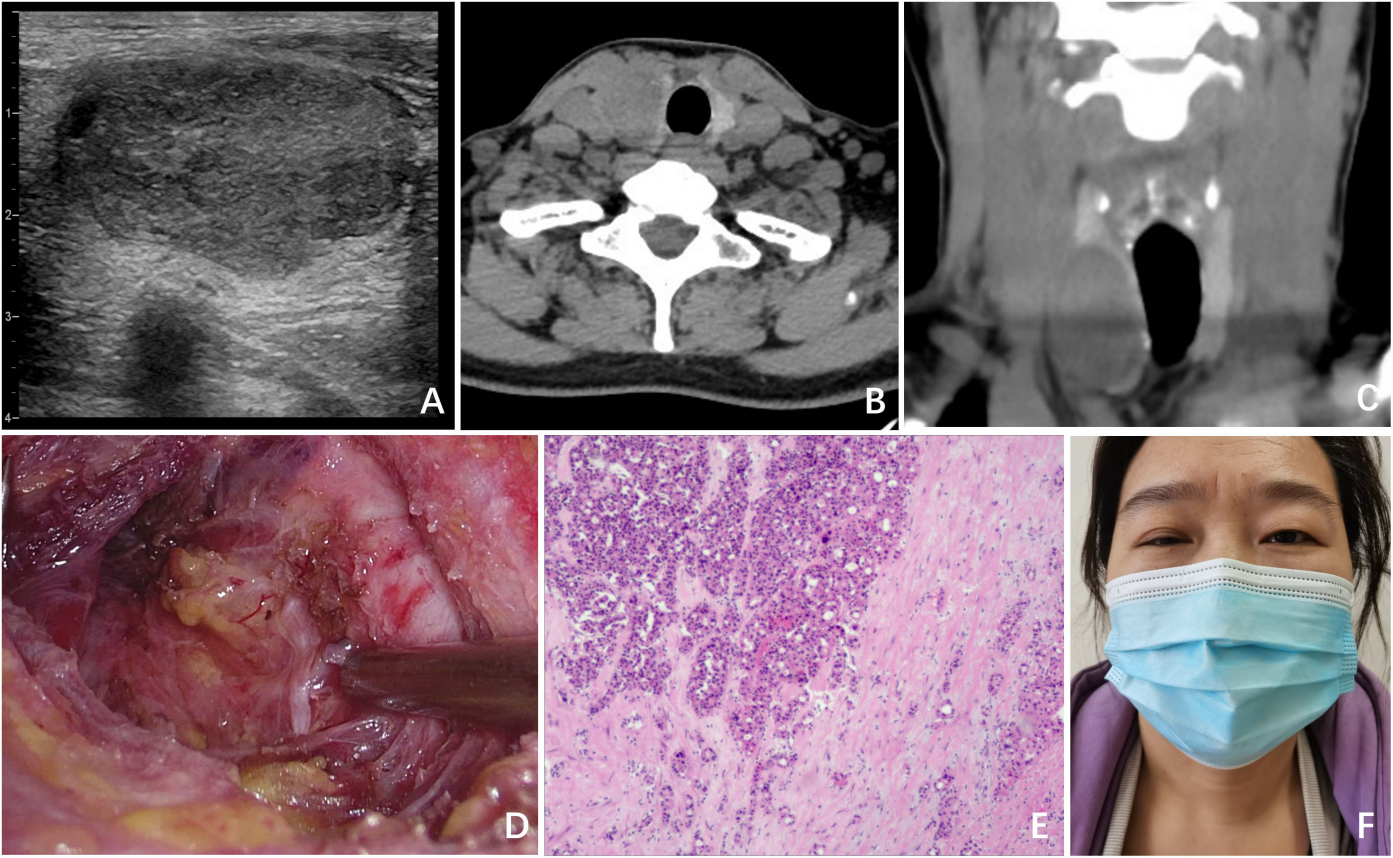
Clinical data of Case 1. **(A)** Ultrasound revealed a nodule in the right lobe, which had low echogenicity and measured 4.0 cm × 2.4 cm × 2.2 cm; **(B)** CT (transverse section) revealed a cystic low-density shadow with clear boundaries in the right lobe of the thyroid; **(C)** CT image of the coronal plane; **(D)** surgical field after UT by ETS; **(E)** postoperative pathology indicated thyroid oncocytic adenoma; **(F)** on POD 1, ptosis of the right eyelid and miosis of the right eye were observed in the patient.

A 43-year-old female patient presented with a 1-month history of an asymptomatic thyroid nodule in the right lobe, which was initially detected through an ultrasound examination. The ultrasound revealed a nodule in the right lobe with low echogenicity, measuring 4.0 cm × 2.4 cm × 2.2 cm. It was classified as TI-RADS 3 (Figure 1A). She had no relevant medical, radiological, or family history of thyroid disease. Upon physical examination, a firm nodule with a diameter of 4 cm was palpable in the right thyroid lobe. A computed tomography (CT) scan revealed a cystic low-density shadow with clear boundaries in the right lobe of the thyroid (Figures 1B, C).

The patient subsequently underwent unilateral thyroidectomy (UT) via an endoscopic bilateral areolar approach (Figure 1D). During the surgical procedure, intraoperative nerve monitoring (IONM) was used to confirm the presence of normal signals from the recurrent laryngeal nerve and vagus nerve. The operation proceeded smoothly, with no complications or unexpected findings. Intraoperative rapid frozen section pathology identified the lesion as a follicular thyroid tumor.

Postoperative pathology further confirmed the diagnosis as a thyroid oncocytic adenoma, with no evidence of capsular or vascular invasion (Figure 1E). On postoperative day (POD) 1, we observed ptosis of the right eyelid, miosis, and anhidrosis on the ipsilateral face in the patient, with no signs of vascular dilatation (Figure 1F). Consequently, a comprehensive ocular examination was jointly carried out by a neurologist and an ophthalmologist. The right pupil displayed noticeable constriction compared to the left pupil (right: 2 mm, left: 4 mm), indicating the presence of anisocoria. Following apraclonidine testing, the right pupil dilated more than the left pupil (right: 3 mm, left: 4 mm) 45 min after the instillation of 0.5% apraclonidine eye drops, indicating a reversal of the previously observed anisocoria. Ipsilateral anhidrosis was observed during the neurological assessment. The degree of pupillary dilation on the affected side is used to determine the lesion of the second - order neuron in the OSP. After ruling out other potential complications, including intracranial lesions, hematoma, dyspnea, inflammation, or vocal cord problems, the patient was diagnosed with HS. Following informed consent from the patient, a treatment plan was implemented, consisting of a 3-day course of intravenous dexamethasone at a dose of 10 mg once daily, and a 6-day course of intravenous mecobalamin at a dose of 0.5 mg once every 2 days. However, following the administration of glucocorticoids and neurotrophic drugs, no improvement in symptoms was noted after 1 week of treatment. Fortunately, at the 6-month follow-up visit, the patient’s HS symptoms had completely resolved, and she expressed satisfaction with the cosmetic outcome of the ETS.

### Case 2

**FIGURE 2:**
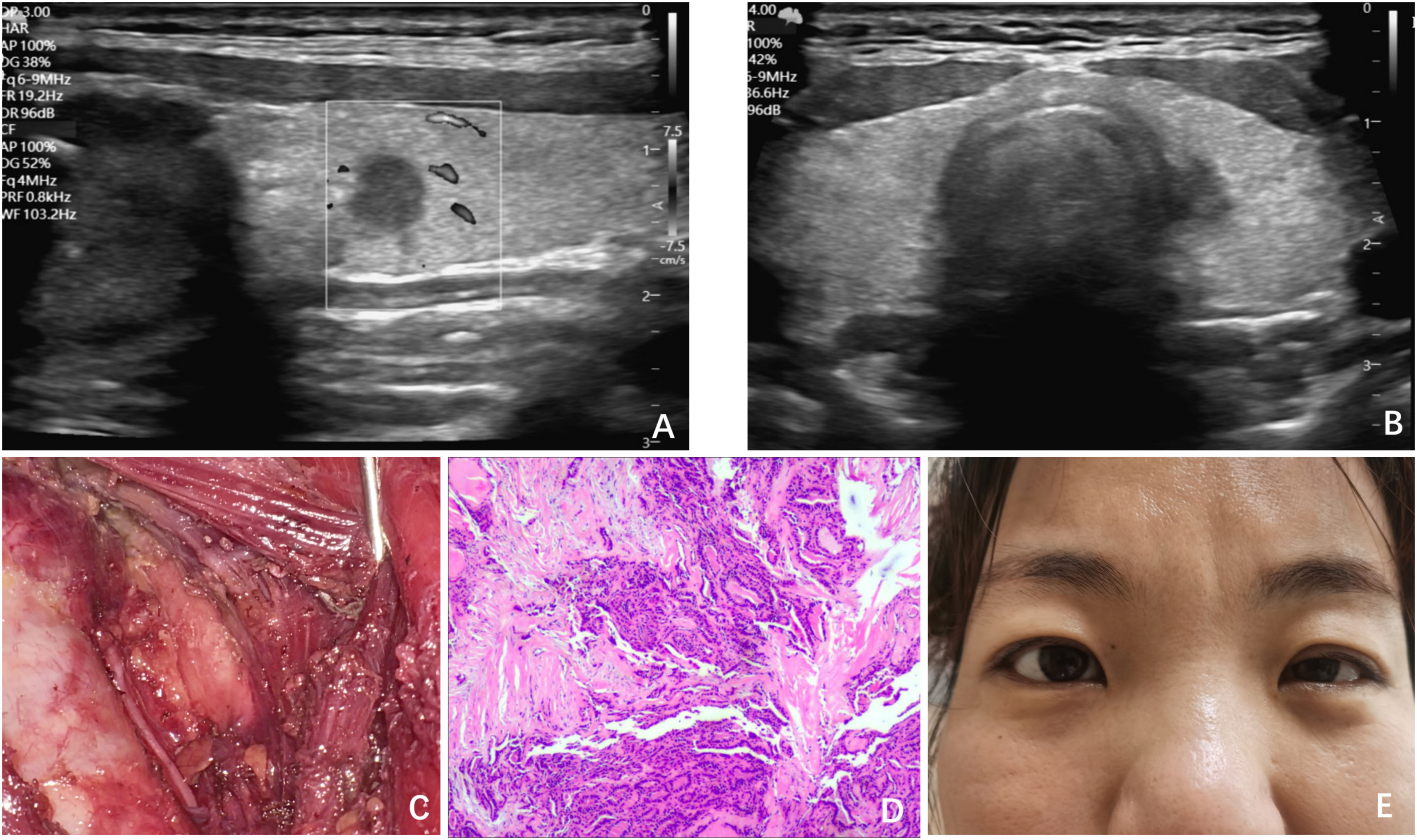
Clinical data of Case 2. **(A)** Ultrasound revealed a low echogenicity nodule, measuring 0.5 cm × 0.5 cm × 0.6 cm; **(B)** ultrasound revealed the nodule adjacent to the trachea in the left thyroid lobe; **(C)** surgical field after UT and CLND by ETS; **(D)** postoperative pathology indicated PTC; **(E)** on POD 1, ptosis of the left eyelid and miosis of the left eye was observed in the patient.

A 36-year-old female patient was referred to our surgical department following an ultrasonographic examination that revealed a low echogenicity nodule, measuring 0.5 cm × 0.5 cm × 0.6 cm, adjacent to the trachea in the left thyroid lobe (Figures 2A, B). FNA biopsy was performed and confirmed the presence of papillary thyroid carcinoma (PTC). The patient’s physical examination, personal history, and family history were all unremarkable. Despite our recommendation for active surveillance of the nodule, the patient strongly preferred surgical intervention. Consequently, with the aim of achieving a satisfactory postoperative neck appearance, she underwent ETS with UT and central lymph node dissection (CLND) (Figure 2C). IONM demonstrated normal nerve function. As anticipated, postoperative pathological examination confirmed the diagnosis of PTC (Figure 2D), but no lymph node metastasis was identified in the central region.

On POD 1, the patient exhibited symptoms, such as ptosis of the left eyelid, miosis, and anhidrosis on the ipsilateral face, and was similarly diagnosed with HS after comprehensive examination (Figure 2E). After excluding other potential complications, we initiated a 6-day course of glucocorticoid and neurotrophic therapy, which resulted in the complete resolution of symptoms.

## Discussion

Horner syndrome (HS) refers to a group of clinical syndromes characterized by nerve paralysis resulting from damage to the cervical sympathetic chain (CSC), a nerve bundle on the OSP, with the primary mechanism underlying HS following thyroid surgery being direct injury to the CSC due to anatomical factors (16). As a rare complication of ETS, HS had an incidence of 0.03%∼0.48% (9, 10, 17–20).

Understanding the anatomical basis of the OSP and CSC can further deepen our comprehension of HS. The OSP, composed of a pathway with three types of neurons, is originates from the central nervous system, traverses the CSC, and ultimately reaches the eye (21). The second–order neurons, which are located in the region where the CSC resides, are significantly correlated with iatrogenic injuries (2). The CSC is located posterior to the carotid sheath, anterior to the longus muscles, inferior to the prevertebral fascia, and is connected to the superior, middle, and inferior cervical ganglia. Excessive traction, dissection, extensive surgical procedures, or complex thyroid surgeries can cause CSC damage, which increases HS risk (22).

ETS has gained widespread application as an alternative to conventional surgery, primarily due to its ability to deliver satisfactory cosmetic outcomes in patients. However, its narrower operating space necessitates stricter requirements for surgical field exposure. When using an endoscope-specific retractor to separate the band muscle, excessive tension compressing the carotid sheath may lead to transient neural damage in the CSC, resulting from consequent hematoma, ischemia, edema and inflammatory response. This mechanism was mentioned in all the aforementioned literature and is the presumed cause of HS in these two postoperative cases. This pathological alteration, resulting in compression of the CSC, is particularly prominent in the early postoperative period following ETS. Over time, as hematoma absorption occurs, ischemia improves, and edema and inflammation subside, the symptoms of HS may gradually resolve. This hypothesis can account for the observation that the onset of HS typically occurs within 3 days after ETS, and during follow-up, the symptoms were completely resolved in most cases.

Energy devices, particularly ultrasonic instruments that provide the dual advantages of cutting and coagulation, have been widely used in various surgical procedures and play a significant role in endoscopic surgery. During ETS, thermal injury caused by ultrasonic instruments is another potential cause of nerve damage, especially in patients with anatomical variations of the CSC. Carlander (23) reported in a rat model study that the local energy effects generated by ultrasonic instruments can lead to neurological dysfunction, and the extent of nerve damage depends on the duration of thermal exposure. Furthermore, other studies have shown that the incidence of temporary recurrent laryngeal nerve paralysis is higher after the use of ultrasonic instruments compared to traditional techniques (24).

The parapharyngeal space is another anatomical location that requires attention. At the C1-C4 levels, the superior cervical ganglion (SCG) which is the largest ganglion in the CSC, is located posterior to the carotid sheath, and is anatomically adjacent to the parapharyngeal space. Cases such as excessive dissection of the upper pole during ETS, which resulted in HS, may be associated with the SCG injury (15). Although the incidence of HS after endoscopic surgery is lower than that after conventional surgery, in the early stages of implementing endoscopic surgery, the incidence remains relatively high (8). Due to a lack of experience, the carotid sheath often needs to be retracted in the limited operative space when dealing with the superior pole of the thyroid, exposing the surgical field (15). Using a retractor to manipulate the carotid sheath risks exposing or damaging the SCG.

In the event of HS occurring after ETS, a differential diagnosis must be conducted in conjunction with departments such as neurology and ophthalmology to exclude potential causes, including intracranial lesions, cervical spinal cord lesions, cervicothoracic tumors, infections, immunologic diseases, or carotid artery lesions, before it can be considered a complication of ETS.

To reduce the incidence of HS during ETS, several preventive measures should be considered. First, minimizing retraction forces on the carotid sheath can help avoid excessive compression of the CSC. Second, avoiding excessive use of energy devices near the carotid sheath is crucial, as thermal injury from ultrasonic or electrocautery devices can lead to CSC damage. Third, recognizing anatomical variations in the CSC is essential, as variations may increase the risk of iatrogenic injury. A recent publication by Tok et al. (25) emphasizes these points in the context of conventional thyroidectomy with central neck dissection, underlining the importance of surgical technique and anatomical awareness in preventing HS. Including this perspective broadens the applicability of our discussion across different surgical approaches.

While IONM was utilized in both presented cases, HS still occurred, suggesting that IONM alone may not be sufficient to prevent injuries to the CSC. IONM primarily monitors motor nerves, such as the recurrent laryngeal nerve and vagus nerve, but does not provide feedback on autonomic fibers like those comprising the CSC (26). Therefore, reliance on IONM without direct visualization or anatomical awareness of the CSC may give a false sense of security regarding nerve preservation during ETS. Surgeons should be aware of these limitations and incorporate direct visualization and anatomical knowledge into their surgical approach to minimize the risk of CSC injury.

The clinical diagnosis of HS is primarily based on the patient’s signs, particularly the asymmetry in pupil size and reactivity, as well as the phenomenon of delayed pupil dilation in dim light. Pharmacologic tests utilizing drugs such as Apraclonidine, Cocaine, and Hydroxyamphetamine can further aid in confirmation of the diagnosis and assist in localizing the lesion site (2). In our cases, Apraclonidine eye drops were used to confirm the diagnosis of HS, revealing a reversal of ptosis and miosis on the affected side, consistent with OSP disruption. Short-term administration of steroids and neurotrophic therapy can promote neuronal repair and help alleviate these symptoms (10, 12, 14). When the symptoms of HS persist for more than 1 year, the probability of full recovery significantly decreases (11). Although HS typically does not affect ocular function, it causes significant cosmetic and psychological distress to patients, particularly after endoscopic surgeries that are intended to enhance appearance. Phenylephrine, cocaine, hydroxyamphetamine, apraclonidine, naphazoline, and oxymetazoline can achieve short-term blepharoptosis correction (27). Corrective surgeries such as tarsoconjunctival mullerectomy combined with levator resection and frontalis sling procedure can alleviate the symptoms of ptosis (28).

## Conclusion

HS is a clinical sign that represents the impairment of the CSC, which is considered to be a rare and non-life-threatening complication following ETS. This complication arises from multiple injury mechanisms. Patients should be fully informed of this risk through detailed preoperative counseling prior to surgery, and the area surrounding the CSC should be carefully assessed and precisely operated to avoid damaging it.

## Data Availability

The raw data supporting the conclusions of this article will be made available by the authors, without undue reservation.
